# In-vivo validation of interpolation-based phase offset correction in MR flow quantification: a multi-vendor, multi-center study

**DOI:** 10.1186/1532-429X-18-S1-O88

**Published:** 2016-01-27

**Authors:** Mark B Hofman, Manouk J Rodenburg, Karin Markenroth Bloch, Beat Werner, Jos J Westenberg, Emanuela Valsangiacomo-Buechel, Robin Nijveldt, Onno A Spruijt, Philip J Kilner, Albert C van Rossum, Peter Gatehouse

**Affiliations:** 1 grid.16872.3a000000040435165XPhysics and Medical Technology, ICaR-VU, VU University Medical Center, Amsterdam, Netherlands; 2 grid.10419.3d0000000089452978Radiology, Leiden University Medical Center, Leiden, Netherlands; 3 grid.16872.3a000000040435165XCardiology, ICaR-VU, VU University Medical Center, Amsterdam, Netherlands; 4 grid.16872.3a000000040435165XPulmonology, VU University Medical Center, Amsterdam, Netherlands; 5 grid.439338.6CMR unit, Royal Brompton Hospital, London, UK; 6 grid.412341.1Division of Cardiology, University Children's Hospital, Zürich, Switzerland; 7 Philips, Lund, Sweden

## Background

Velocity offset errors in phase contrast MR imaging are a known problem in clinical assessment of flow volumes in vessels around the heart. Earlier studies showed that offset errors can be clinically relevant over different systems, and are not consistently reduced by protocol optimization [1, 2]. Correction methods using phantom measurements are time consuming, and assume reproducibility of the offsets which is not the case for all systems [3]. An alternative solution is to correct the *in-vivo* data in post-processing, interpolating the velocity offset from stationary tissue within the field-of-view [4]. Complementing a previous single center study [5], this study aims to validate this offset correction *in-vivo* in a multi-vendor, multi-center setup.

## Methods

Data from six 1.5 T MR systems were evaluated, with two systems from each of the vendors GE, Philips and Siemens. Aortic and main pulmonary artery 2D flow studies were acquired during routine clinical or research examinations. Each site used their local standard scan protocol, with limited spatial wrap around, retrospective ECG gating, and without other phase offset correction methods (i.e. Philips LPC filter off). Within the same day a phantom measurement was obtained with identical acquisition parameters.

To verify the phantom acquisition, a ROI at stationary tissue in the thorax wall was placed and compared between *in-vivo* and phantom measurements. Only studies with an agreement within 0.6 cm/s between these check ROIs were used in the following analysis.

Interpolated velocity offsets from the *in-vivo* data were calculated using the algorithm of Walker *et al.* [4, 5], after manually excluding regions of spatial wraparound. Correction performances of different spatial orders of interpolation planes were tested. The calculated velocity offsets obtained in a ROI at the location of the vessel were compared to the offsets measured in the phantom scan. The impact of correction was assessed on cardiac output.

## Results

A total of 123 flow measurements in 80 subjects were included (56 MPA, 67 AO). At the thorax wall the agreement between *in-vivo* and phantom was -0.2 ± 0.6 cm/s. 27 studies were further excluded due to too large deviation of the phantom scan at the thorax wall. Before correction the offset at the vessel (as assessed in the phantom) was -0.7 ± 1.4 cm/s, and resulted in a -6 ± 16% error in cardiac output. The optimal order of the correction plane was 1^st^ order, except for one GE system (system 6) at which a 2^nd^ order plane was required. After application of the interpolation correction the remaining offset velocity was 0.0 ± 0.5 cm/s and 0 ± 5% error in cardiac output.

## Conclusions

This study shows that interpolation-based offset correction reduces the offset with comparable efficacy as phantom measurement phase offset correction without the time penalty imposed by phantom scans.

**Figure 1:**
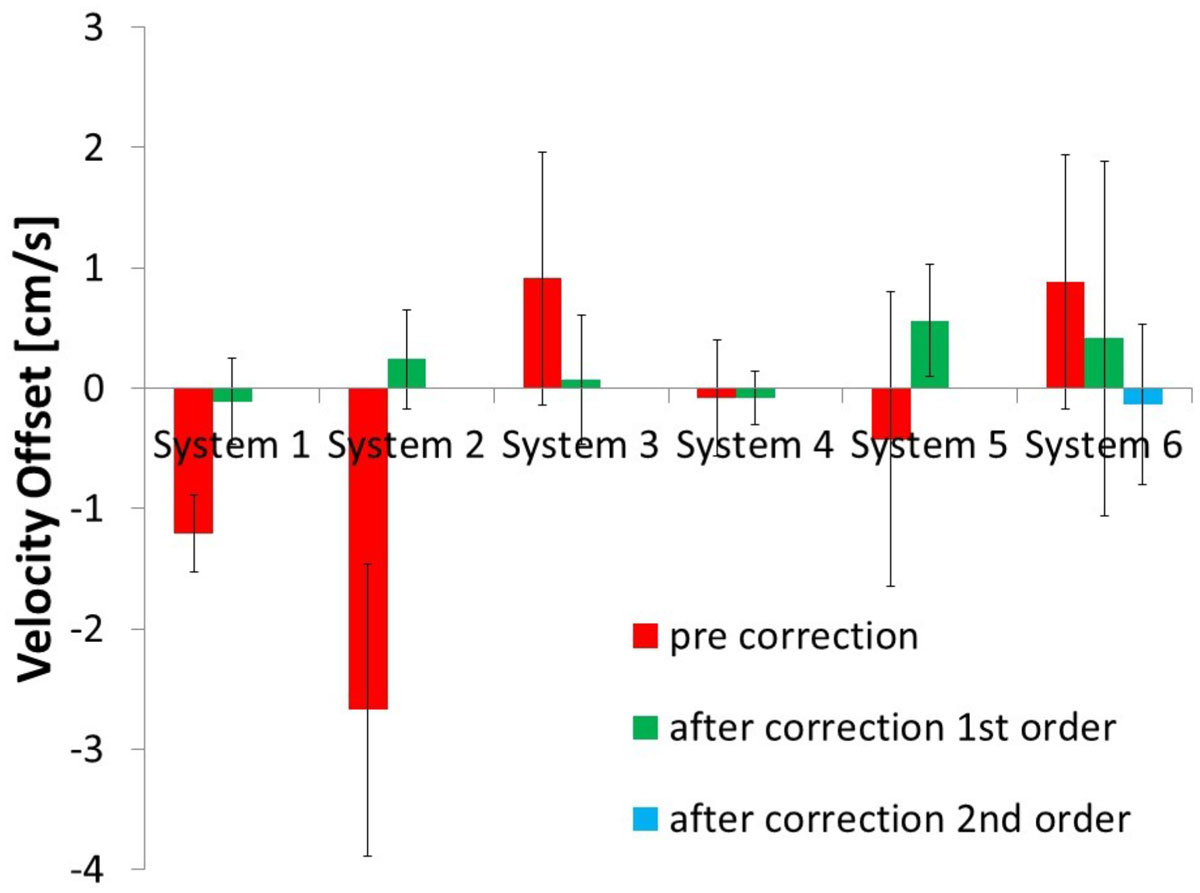
**Velocity offset at aorta and main pulmonary artery before and after offset correction (mean and SD per MR system)**.

**Figure 2:**
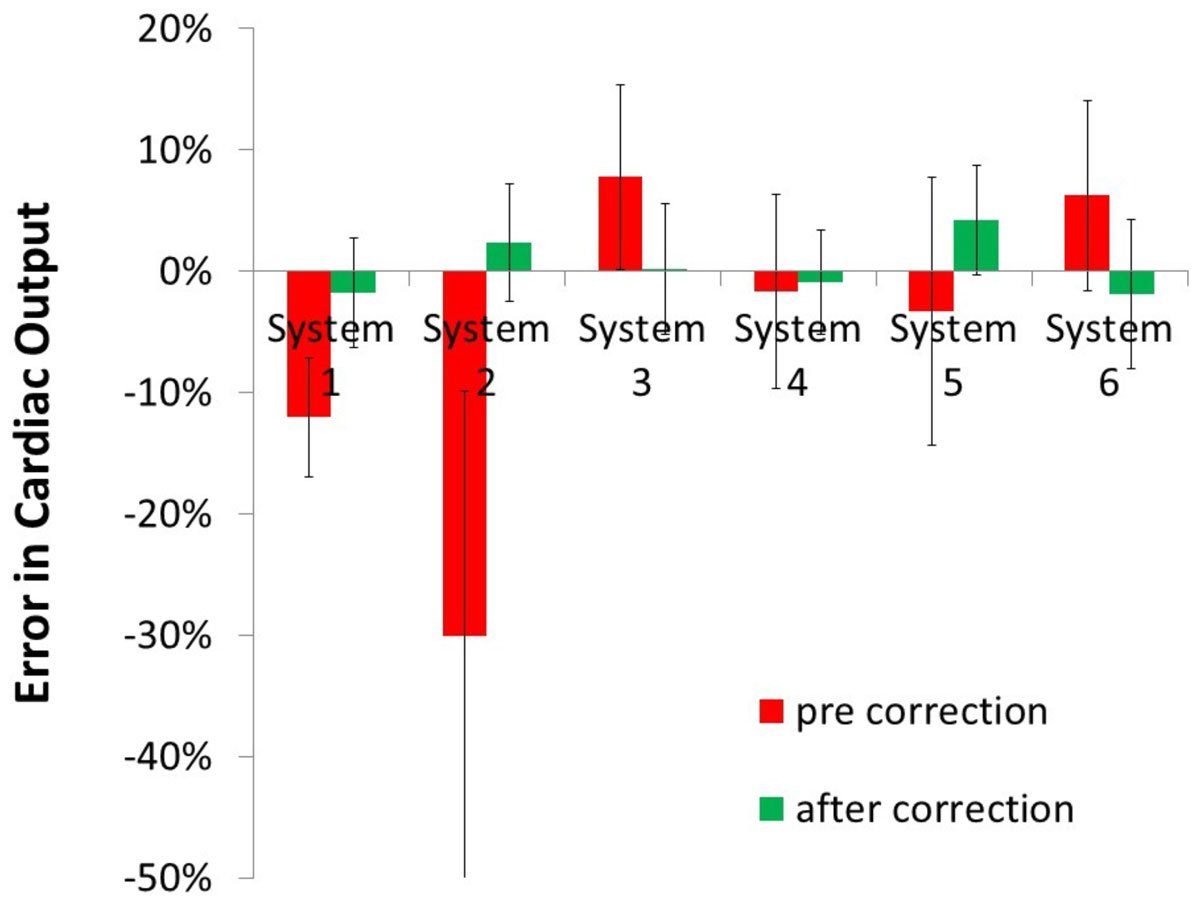
**Error in cardiac output before and after offset correction (mean and SD per MR system)**.
